# Simulation of nerve fiber based on anti-resonant reflecting optical waveguide

**DOI:** 10.1038/s41598-022-23580-4

**Published:** 2022-11-11

**Authors:** Marzieh Omidi, Mohammad Ismail Zibaii, Nosrat Granpayeh

**Affiliations:** 1 Center of Excellence in Electromagnetics, Faculty of Electrical Engineering, K. N. Toosi University of Technology, Tehran, Iran; 2 Laser and Plasma Research Institute, Shahid Beheshti University, Tehran, Iran

**Keywords:** Biological techniques, Optics and photonics

## Abstract

Light and optical techniques are widely used for the diagnosis and treatment of neurological diseases as advanced methods. Understanding the optical properties of nervous tissue and nerve cells is vital. Using light sources in these methods raises significant challenges, such as finding the place of light transmission in nerve fibers that could be an appropriate substrate for neural signaling. The myelinated axons are a promising candidate for transmitting neural signals and light due to their waveguide structures. On the other hand, with the emergence of diseases such as multiple sclerosis and disorders within the production and transmission of nerve signals, because of the demyelination, understanding the properties of the myelinated axon as a waveguide is obtaining additional necessity. The present study aims to show that the myelinated axon’s refractive index (RI) profile plays an essential role in transmitting the beams in it. According to the nerve fiber, RI profile and its similarity to depressed core fiber with lower RI of the core compared to the cladding, the behaviors of the nerve fiber based on anti-resonant reflecting optical waveguide structure are investigated by taking into account the realistic optical imperfections. Light launching to the myelin sheath and axon is shown by introducing the axon and myelin sheath as a waveguide in the presence of both axon and myelin with bends, myelin sheath variation, and node of Ranvier.

## Introduction

The brain’s complex structure has caused a great deal of interest in researching neuronal communication, aiming to understand the signals and their integration into neural functions at the highest level^1^. As part of its function, the nervous signaling system plays a crucial role^2^ and the most well-known neural communication signal results from electrochemical interaction between the ion channels in the cell membrane^3^. Many fundamental questions remain unanswered^4^, such as how the brain^5^ generates the mind and consciousness. Recently, experimental and theoretical evidence has shown that biophotons^6^ may play an effective role in the transmission and processing of neural signals and help to understand the high performance of the nervous system^7^. Much evidence confirms that mitochondrial oxidative metabolism is the primary source of biophoton production in neurons^8^. Meanwhile, optogenetics as an optical tool has been developed to access deeper areas of the brain to understand the nervous system and neurons^9–11^, based on genetic manipulation and using unique wavelengths for optical stimulation of neural cells^12,13^. Questionably, whether light sources used in optical techniques, such as optogenetics, can propagate and be directed in a specific path inside the nerve cells^14^. To this end, the trajectory of biophotons and light rays has been studied from an engineering and optical point of view, and the myelinated axons have been introduced as highly efficient waveguides for conducting light rays and biophotons^1,14^.

Axons are a suitable substrate for photon signaling due to their proper configuration, which contains many microtubules and mitochondria^15,16^. Due to axons’ waveguiding nature and their essential role in transmitting neural information, they have received much attention as a vital component of the nerve cell and nervous system. Thus, the interaction of light and neurons has been investigated by assuming biophotons or light rays from external sources in the myelinated axon.

Additionally, some researchers have focused on inversed index fibers in recent years, such as depressed core fibers (DCFs), defined by a three-layer fiber, including a low-index core and a high-index cladding, surrounded by air^17^. In contrast with conventional step-index fibers, where light is guided by total internal reflections (TIRs) in the high-index core region, inverse-index fibers can guide light by other mechanisms such as the anti-resonant effect in the low-index core, which can be modeled by an anti-resonant reflecting optical waveguide (ARROW) ^18^ acting as a Fabry–Perot resonator (FPR)^19^, which allows the anti-resonant light to be reflected back while allowing forward transmission of the resonant light. FPRs usually exhibit resonances over a narrow band of wavelengths, whereas their anti-resonances are quite spectrally broad^19,20^.

The refractive index (RI) profile of myelinated axons is very similar to DCF. This study uses a full-wave 3D computational nerve fiber model based on the ARROW model to analyze photon transmission inside the myelinated axon. The obtained results revealed that the condition and location of light coupling to the myelinated axon are directly related to the ARROW structure. In this situation, the effects of nerve fibers' physical factors, including the size of the myelin layer, the node of Ranvier (NR) dimensions, and the curvature of the myelinated axon on the photonic signaling, are assessed. This approach can be a preliminary neural model that provides the basis for new studies in the field of cellular messaging and deep brain stimulation based on the concepts of nanophotonic communication. ARROW-based nerve fiber structure can help to introduce a biosensing method based on fiber optic for the optical and label free study of neural cell structure can be used to measure optical properties of nervous systems and early detection of neurological diseases such as MS. The review of literatures on transmitting light in nerve fibers is summarized in Table 1.

**Table 1:** A review of the researches on transmission of light in nerve fiber.

Mechanism	Source	Size	Optical imperfections	Results	Ref
Myelin sheath as a waveguide of biophotons	Biophoton @ Infrared and visible	*g-ratio* = 0.6 L_axon_ = 100 µm D_axon_ = 0.6–3 µm	Bending Variation of cross section of myelinated axon	The myelinated axon as a waveguide guides photons only in myelin sheath Presence of bending, varying cross-sectional area and increasing the diameter of the nerve fiber, reduces light transmission	^7^
Ranvier node as Bio-Nano antennas	Eectromagnetic radiation @ Infrared and visible	*g-ratio* = 0.78 L_axon_ = 100 µm D_axon_ = 0.57 µm	NR NR as antenna	NR of myelinated axons as a nanoantenna array system Radiate optical waves propagating through the myelinated axon Myelinated axon acts as waveguide	^1^
Nerve fibers as a waveguide	Biophoton / external light source @ visible	*g-ratio* = 0.7 L_axon_ = 27 µm D_axon_ = 0.4 µm	NR demyelination Myelin as lossy and lossless media	Axon acts as a waveguide Assessed myelin sheath as lossless and lossy model Propose a mechanism based on nanoparticles to repair photon transmission inside demyelinated nerve fibers	^14^
Label-free nanoscale optical metrology on Myelinated axons in vivo	Spectrally scanned white-light laser @ visible	D_axon_ = 0.5 µm Myelin thickness: 18-48 nm	Myelin as Multi-layered NR Variation of axon size Variation of number of myelin layers	The Multi-layered features of myelin act as a thin film around the axon fibers Spectral reflectometry (SpeRe) is able to nanoscale imaging of myelinated axons in their natural living state SpeRe can be used to investigate osmotic swelling and traumatic brain injury by determining the degree of myelination, wavenumber period for axon diameter, and spectral shift for myelin swelling	^21^
Myelin as coil inductor and cell membrane as a piezoelectric	Two voltage sources	D_axon_ = 1.2 µm Myelin thickness = 10 nm spacing between the two layers = 4 nm with 15 rounds	Myelin as Multi-layered NR	Myelin sheath acts as a coil inductor to generate a magnetic field The coil inductance of myelin and the piezoelectric effect of cell membrane explains the measured mechanical wave and the spiraling of the myelin sheaths in neurons	^31^
Müller cells act as a waveguide to improve day vision	Broad spectral source @ Visible	Müller cell length: 130 nm proximal cup of Müller cell :12 µm in diameter	Not available (N/A)	The Müller cell, performing as a wavelength-dependent optical fiber In Müller cells, the wavelength of incident light is sorted so that the wavelengths suitable for cones are directed to the cones, while those are more suitable for rods leak outside the Müller cells and reach the surrounding rods	^32^
Mitochondria as a waveguide	Electromagnetic radiation @ Visible	N/A	N/A	Microtubules and mitochondria function as optical waveguides due to their higher refractive index compared to cytoplasm Mitochondria as an optical multi-layer system Mitochondria emit chemiluminescent light, and light can be guided along with the mitochondrial network	^33^
Nerve Fiber as an Anti-Resonant Reflecting Optical Waveguide	External light source @ visible	G-ratio = 0.7 L_axon_ = 27 µm D_axon_ = 0.4 µm	Bending NR	The myelinated axon as a waveguide Beams propagation in both axon and myelin sheath, depending on the source launching to myelinated axon In anti-resonant condition the myelin acts as Fabry-Pérot and light propagate in core In resonance wavelength if light launches to myelin, total internal reflection will happen	This paper

The other parts of this study are organized as follows: Section "Analysis Methods" explains the analysis methods. The results are given in section "Results and discussion". The study is concluded in section "Conclusion".

## Analysis methods

This study used CST Microwave Studio to simulate the light transmission diagram. A waveguide port was placed at the beginning and end of the neuron with Gaussian stimulation to obtain the percentage of light transmittance in different states. Open and add boundaries (in CST) were used with half wavelength space between the boundaries and the structure.

For conduction modes calculation, this research has used the finite element method (FEM) numerical analysis of the COMSOL Multiphysics software to obtain the electric field component in axon and myelin^1^. The electromagnetic beam envelopes (EMBE) model was used to simulate the propagation of electromagnetic beams efficiently unidirectional and bidirectional. In order to compute guided modes in the EMBE model, boundary mode analysis was applied. Input and output ports (boundary) are located once on the axon and once on the myelin.

Unfortunately, no data related to the imaginary part of the RIs of different parts of the nerve fiber were available, and since simulated short lengths of the nerve fiber were considered, we assumed that the nerve fiber losses were negligible.

## Results and discussion

### Nerve fiber model

**Figure 1:**
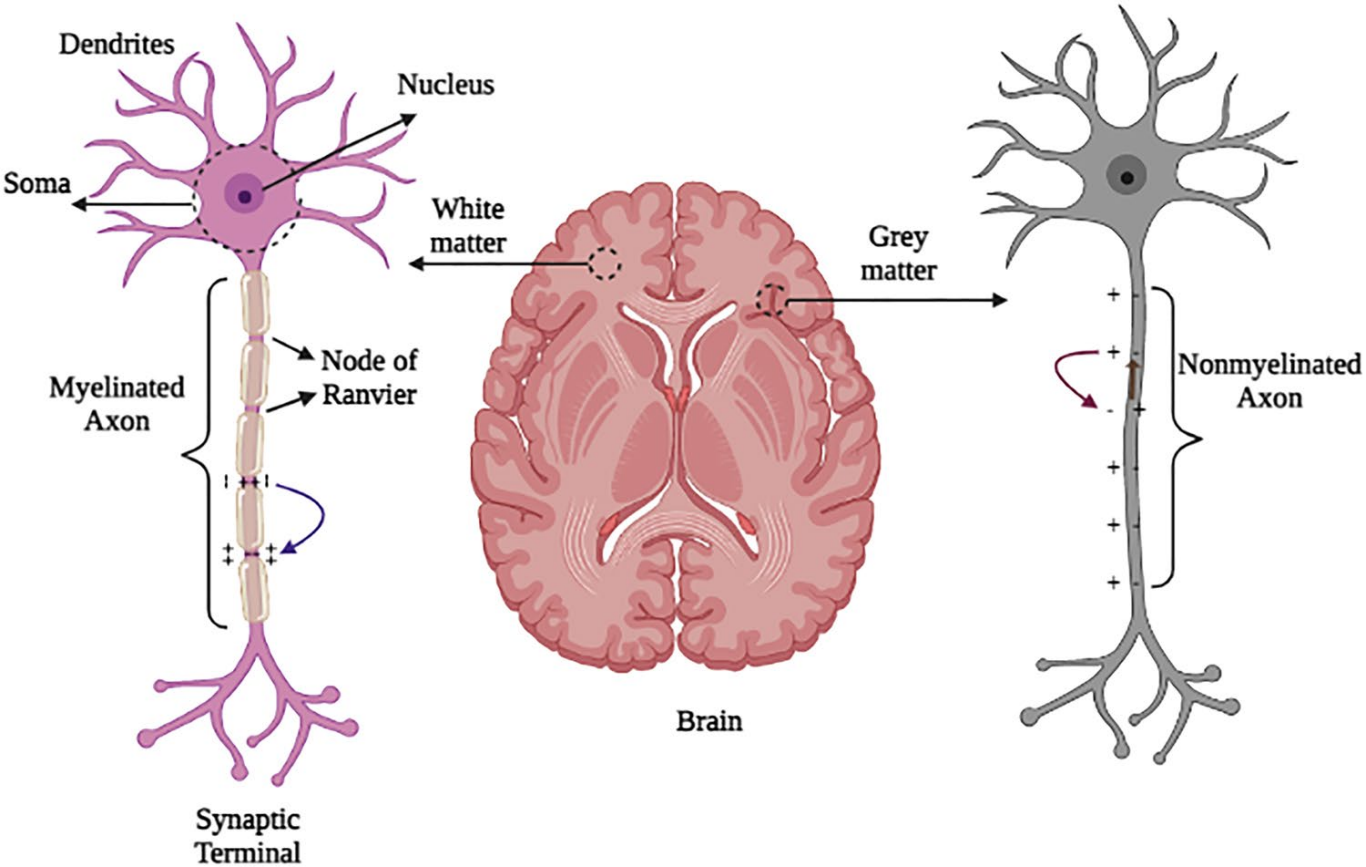
Schematic structure of two different neurons in the brain’s white matter and gray matter. Electrical variations that occur in stimulated (**a**) myelinated (salutatory conduction) and (**b**) nonmyelinated axons.

Nervous tissue is made up of neurons and neuroglia cells. The neuroglia cells support neurons but do not participate in messaging. However, the neurons are excitable cells that create, conduct, and transmit neural messages to other cells^2,3^. A schematic diagram of a neuron is shown in Fig. 1. The cell body, dendrites, and axons are the three major components of a neuron. Axons are the nerve fibers that carry messages from a cell to the synaptic terminal^3^. As shown schematically in Fig. 1a, axons are covered in a myelin sheath, which acts as an insulator and increases signaling speed ^21^. Action potentials (APs) in myelinated axons can be produced only at the NR and jumped from one node to another in a process known as saltatory conduction. This is a faster mechanism than what is found in nonmyelinated fibers^3^ in Fig. 1b.

**Figure 2:**
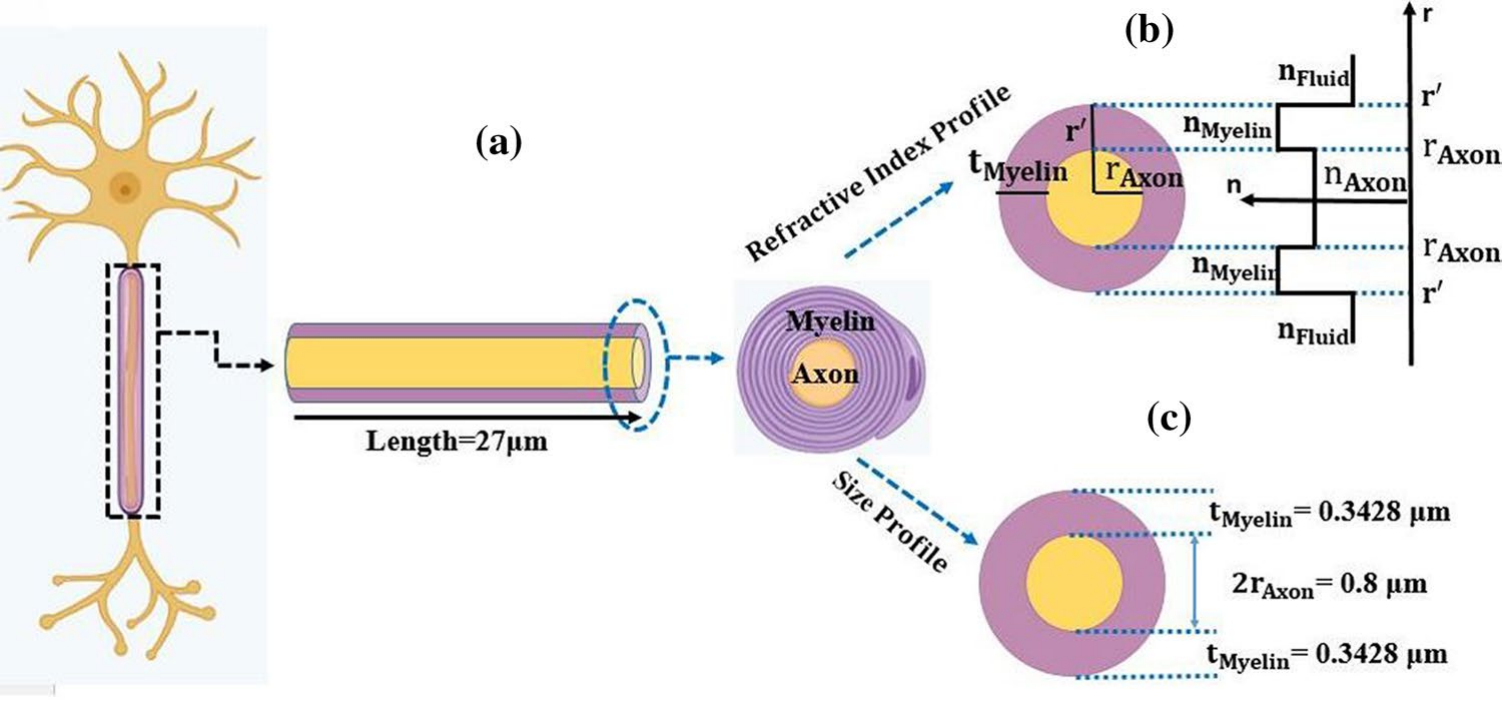
Schematic of structural and optical characteristics of a nerve fiber, (**a**) longitudinal cross-section circular cross-section, (**b**) RI profile and (**c**) the size of Myelin sheath and Axon when *g-ratio* = 0.7.

The nerve fibers act as optical fibers and transmit the light. The RIs of axons, myelin sheath, and extracellular fluid were 1.38, 1.44, and 1.34, respectively^1,7^. The RI profile of the myelinated axon in terms of the radius is shown in Fig. 2. The RI of the axon region ${n}_{Axon}$ was smaller than the RI of the myelin sheath ${n}_{Myelin}$ but greater than the RI of extracellular fluid, ${n}_{Fluid}$, as ${n}_{Fluid}{{<n}_{Axon}<{n}_{Myelin}}$.

The myelinated axons have the same RI profile as DCF. As a result, the principles that describe DCF fiber can be used to characterize myelinated axon behavior. An ARROW model can be used to explain DCF^18^. In the ARROW model, the high-index layers can be considered as FPR, and incident ray energy may be substantially reflected in the core region at anti-resonant wavelengths, and as a result, anti-resonant core modes are formed^18,19^. As explained earlier in Ref.^18^, the modes with ${n}_{air}<{{n}_{eff}={n}_{core}sin{\theta }_{incident}<n}_{core}$ are reflected at the inner boundary in the core and totally reflected at the outer boundary of the clad, where ${n}_{air}$, ${n}_{core}$ and ${\theta }_{incident}$ are RI of air, core, and incident angle of light launched into the core, respectively. Also, the DCF can support the annular-like cladding modes with ${n}_{core}<{{n}_{eff}={n}_{clad}sin{\theta }_{incident}<n}_{clad}$ and guide them to the cladding by total internal reflecting phenomenon^18,24^, where ${n}_{clad}$ is the RI of cladding and ${\theta }_{incident}$ is the incident angle of light launched to the cladding^18^.

**Figure 3:**
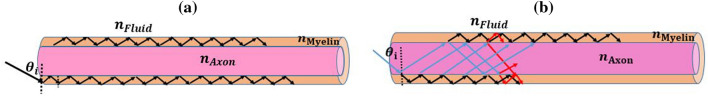
Schematic view of myelinated axons, (**a**) TIR in myelin sheath under conditions ${n}_{Axon}<{{n}_{eff}<n}_{Myelin}$ and (**b**) myelinated axons as FPR under condition ${n}_{Fluid}<{{n}_{eff}<n}_{Axon}$.

Due to this similarity, the ARROW model was studied in two different positions of source light radiation to nerve fiber during this hypothesis. Figure 3 schematically shows the guided modes in myelin and axon with light launched to the myelin and axon, respectively.

With light launched to the myelin sheath, the myelinated axon can support the modes with ${n}_{Axon}<{{n}_{eff}<n}_{Myelin}$ guided by the TIRs at the inner and outer boundaries of the myelin region, as shown in Fig. 3a. When light is launched to the axon, the myelin sheath acts as an FPR, and the modes with ${n}_{Fluid}<{{n}_{eff}<n}_{Axon}$ tend to be totally reflected through the axon and partially reflected in myelin, as shown in Fig. 3b.

Different values for the length and radius of the myelinated axon have been measured ^1,7,14^. Besides, *g-ratio* was expressed as a relationship between the radius of the axon and the thickness of the myelin sheath according to Eq. 1. The *g-ratio* for different nerve fibers varies between 0.6 and 0.8^7,14^. Therefore, according to Ref.^14^, the length of the myelinated axon was roughly 27μm^14^. The axon radius is 0.4μm^14^, and the myelin thickness is obtained through Eq. (1)^7^:

1 $$g{\text{-}}ratio=\frac{2{r}_{axon}}{2{r}_{axon}+2{t}_{myelin}}$$

where ${r}_{axon}$ and ${t}_{myelin}$ were axon radius and myelin thickness, respectively. The *g-ratio* was the ratio of the axon radius (${r}_{axon}$) and the outer radius of the myelin sheath (${r}_{axon}+{t}_{myelin}$), which was assumed to be 0.7 in simulations.

The effects of bending, myelin sheath variation, and the NR on optical properties and light transmission in both cases of the light launched into the myelin sheath and axon were investigated in the following subsections. Also, these imperfections' effect on the ARROW's optical properties was studied.

About the studied wavelength range, the longest wavelength seen from biophotons activities was 1300 nm, and the shortest wavelength was considered 300 nm^7,8,14^ due to the absorption of nerve fibers close to these wavelengths. Also, the optogenetic technique used specific light wavelengths such as 473 nm^11^, 540 nm, and 630 nm^12^ as an optical tool to control neurons’ activities^11^ in which all optogenetic wavelengths were between 400 and 700 nm^21^. Considering the intersection of these two ranges of wavelengths, and according to Ref.^14^, the nerve fiber was simulated in the wavelength range of 300 to 900 nm.

### Effect of myelin sheath thickness on light transmission

The thickness of the myelin sheath was not constant throughout the myelinated axon^21^. The *g-ratio* parameter was varied between 0.6, 0.7, and 0.78 to study the effect of myelin sheath thickness on optical properties, while the axon radius was supposed to be 0.4μm^14^. As previous studies have shown, variation in the thickness of the myelin layer affects the transmission of light and photons^7,14,21^. In this study, the effect of variation in the thickness of the myelin sheath on the ARROW structure has been investigated.

**Figure 4:**
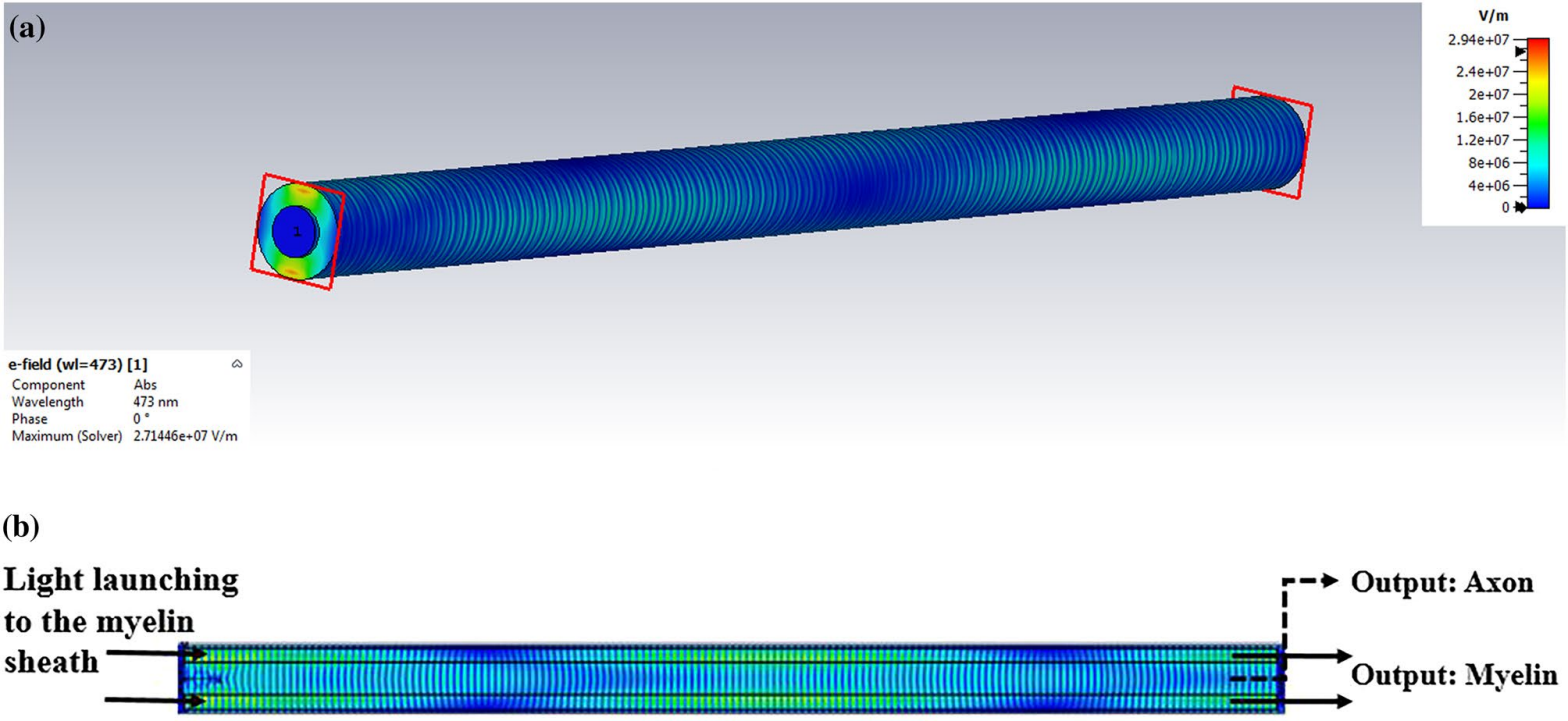
Launching of light to the myelin sheath for *g-ratio* = 0.7. Distribution of the electric field at (**a**) the transverse and (**b**) the longitudinal cross-sections of the myelinated axon.

Foremost, the light was launched into the myelin sheath to investigate the effect of the myelin sheath thickness on optical properties. Figure 4a shows the nerve fiber and its transverse cross-section. Figure 4b indicates the distribution of the electric field at 473 nm as a common wavelength in optogenetic applications. As shown in Fig. 4, if the light is launched in to the myelin sheath, most of the electric field passes through the myelin sheath. Therefore, according to the definition of the structure of ARROW^18^, it can be concluded that the TIR phenomenon occurs in the myelin, similar to DCFs.

**Figure 5:**
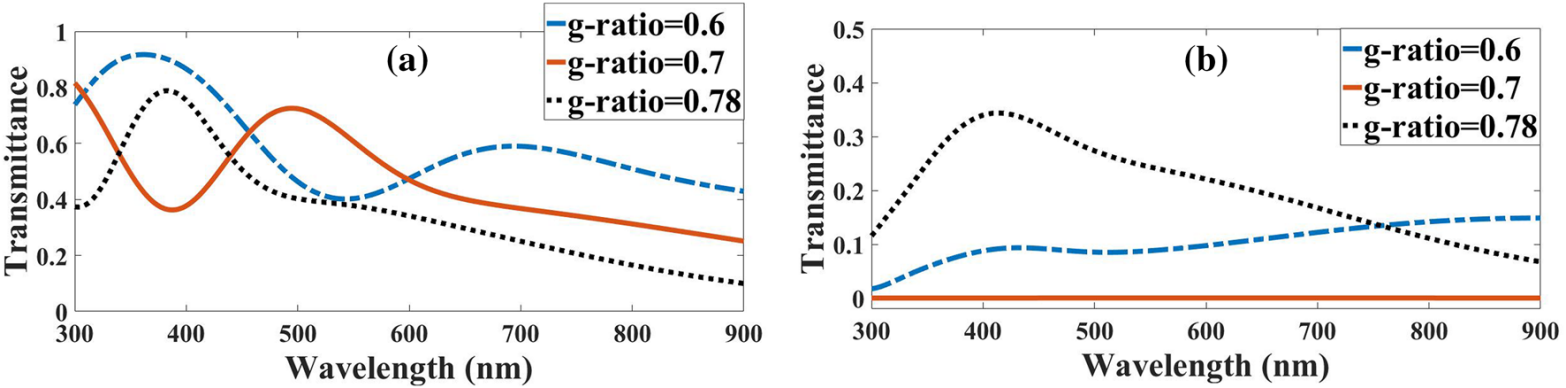
Light transmission spectra for different *g-ratios* at output of the (**a**) myelin sheath and (**b**) axon, when light is launched to the myelin sheath.

The two statuses of outputs from the myelin and the axon by launching the light to the myelin are shown in Fig. 4b. Figure 5a shows the output from the myelin sheath by decreasing the *g-ratio* from 0.78 to 0.7 and 0.7 to 0.6 or increasing the thickness of the myelin sheath, the rate of light transmission in the myelin sheath with a red-shifted increase. The obtained results revealed that with a decline in *g-ratio*, the orders of magnitude transmitting for typical wavelengths compared to *g-ratio* = 0.78 would increase, as shown in Table 2.

**Table 2:** Orders of magnitude transmitting for typical wavelengths in *g-ratio* = 0.7 and *g-ratio* = 0.6 compared to a *g-ratio* = 0.78 when light launched to Myelin in Fig. 5a.

Imperfection	Wavelength (nm)			
	300	473	540	900
*g-ratio* = 0.7	2.1	1.6	1.58	2.5
*g-ratio* = 0.6	1.9	1.28	1.04	4.2

In addition, this feature can be used for spectroscopy in which information from the thickness of the layers or diagnosis of the demyelination complication of the myelin sheath, which disrupts neural messaging, ^14^ were obtained.

Spectral characteristics of the nerve fiber showed a downward, which is based on the fact that the wavelength spectrum approaches the cut-off wavelength of the nerve fiber. Before the cut-off wavelength, the myelin sheath’s propagation was multi-modes; after that, it was single-modes ^14,22^. A broadening was also observed in spectral responses that result from the multi-path dispersion of a fiber^23^.

As shown in Fig. 5b, the output from the axon is the complement of Fig. 5a. In other words, as the transmission in the myelin decreases, more conduction modes pass through the axon. When the light is launched into the myelin, due to the TIR phenomenon, all the rays and conduction modes will expectedly be directed inside the myelin sheath, which has occurred well at *g-ratio* = 0.7.

**Figure 6:**
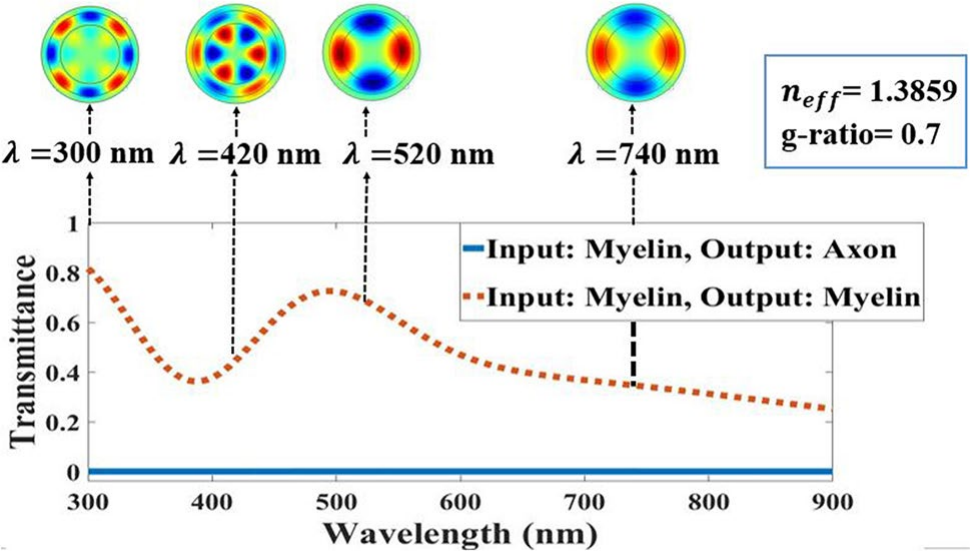
Light transmission spectra in output of the axon and myelin at *g-ratio* = 0.7 with guided modes in myelin sheath in 300 nm, 420 nm, 520 nm, 740 nm wavelengths.

Since the TIR phenomenon was affected by the thickness of the myelin sheath, the best thickness was for *g-ratio* = 0.7, during which the TIR phenomenon occurs. Figure 6 demonstrates the light transmission in the axon and myelin.

As mentioned earlier, in the DCF and ARROW model, if ${n}_{\mathrm{core}}<{{n}_{eff}={n}_{clad}sin{\theta }_{\mathrm{incident}}<n}_{\mathrm{clad}}$ , then the TIR phenomenon occurs and if ${n}_{\mathrm{air}}<{{n}_{eff}={n}_{\mathrm{core}}sin{\theta }_{\mathrm{incident}}<n}_{\mathrm{core}}$ , then the myelin sheath acts as FPR. As a result, the TIR phenomenon occurs in the myelin sheath if effective RIs are in the range of ${n}_{axon}<{{n}_{eff}<n}_{myelin}$. The next step is to validate it by calculating the guided modes in the circular cross-section of the nerve fiber. Also, the distributions of the intensities of the electric field in the x-direction in four different wavelengths of 300 nm, 420 nm, 520 nm, and 740 nm, calculated for an effective RI of 1.3859, while the light is launched to the myelin sheath are shown in Fig. 6.

The defined guided modes of light transmission spectra in myelin in Fig. 6 clearly show how light transmits through the myelin and axon. For example, at 300 nm, the guided modes are confined in a myelin sheath because the light transmission spectra show 80% transmittance through the myelin. However, at 420 nm, guided modes are passed in the axon and myelin. Besides, the light transmission spectra illustrate 45% transmittance in myelin at this wavelength. This means that the numbers of guided modes either lose or pass through the axon, although according to light transmission spectra of the axon (blue line), guided modes in the axon were negligible.

**Figure 7:**
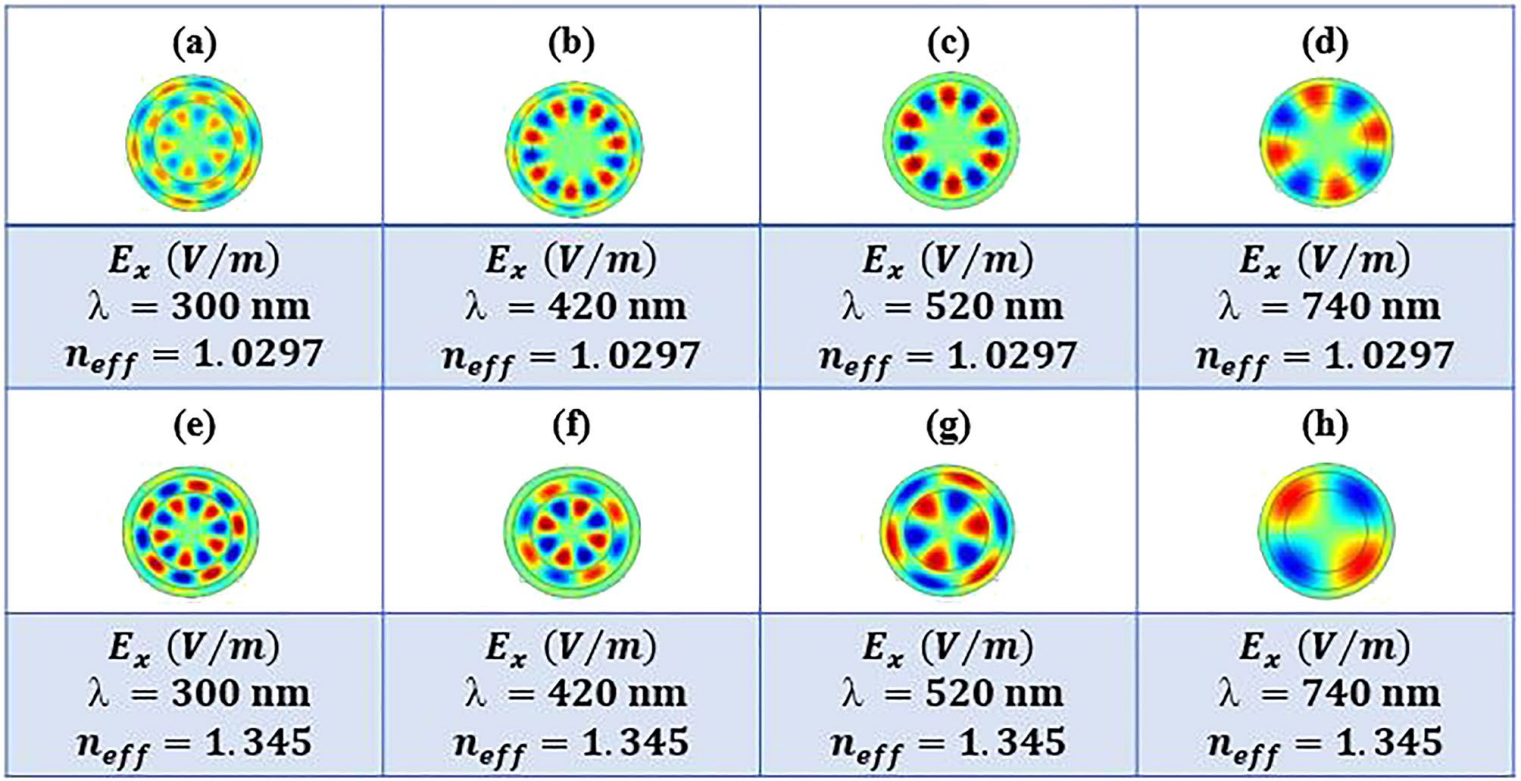
Electric field component ${E}_{x}$ of nerve fiber at 300 nm, 420 nm, 520 nm, and 740 nm wavelengths for different effective RIs, while light is launched to the myelin sheath.

The conduction modes of the nerve fiber are calculated assuming the light is launched only to the myelin sheath. Obtained result are shown in Fig. 7 for different wavelengths and effective RIs. As the characteristics of the conduction modes were more inclined to be guided in the myelin sheath and were entirely consistent with the light transmission diagrams in Fig. 6, it can be concluded that the TIR phenomenon occurs in the myelin sheath.

**Figure 8:**
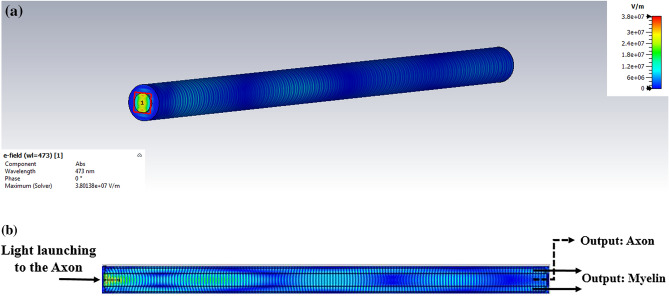
Distributions of electric field when light is launched to the axon, at the (**a**) circular and (**b**) longitudinal cross-sections of the myelinated axon.

Light is launched to the axon, the myelin sheath thickness affects optical properties**.** The nerve fiber can act as an ARROW structure in anti-resonance wavelength with light launching to the axon. Figure 8a,b show the field distributions of the myelinated axon’s circular and longitudinal cross-sections when the light was launched into the axon. In the ARROW model, the myelin sheath can act as FPR, which allows the anti-resonant light to be back-reflected while allowing forward transmission of the resonant light^18,24^ as shown in Fig. 8b, and as a result, anti-resonant core modes are formed^18,19^.

**Figure 9:**
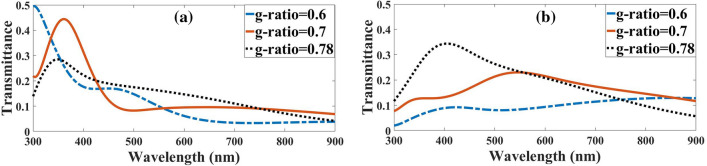
Light transmission spectra to the outputs of (**a**) axon and (**b**) myelin sheath for different *g-ratios*.

Figure 9a,b showed the axon and myelin sheath output spectra for different *g-ratios*, respectively. As shown in Fig. 9a, by decreasing the *g-ratio* from 0.78 to 0.7 and 0.7 to 0.6 while consequently increasing the thickness of the myelin sheath, the rate of light transmission in the axon only increased in lower wavelengths, which was 300 nm to 345 for *g-ratio* = 0.6 and 300 nm to 433 nm for *g-ratio* = 0.7. After these wavelengths, the decline in transmissions was negligible. The orders of magnitude of the transmission for typical wavelengths in *g-ratio* = 0.7 and *g-ratio* = 0.6 compared to a *g-ratio* = 0.78 when the light was launched to the axon, as shown in Table 3.

**Table 3:** Orders of magnitude the transmission for typical wavelengths in *g-ratio* = 0.7 and *g-ratio* = 0.6 compared to a *g-ratio* = 0.78 when light launched to Axon in Fig. 9 (a).

Imperfection	Wavelength (nm)			
	300	473	540	900
*g-ratio* = 0.7	1.5	1.6	1.6	1.6
*g-ratio* = 0.6	3.5	0.9	1.04	1.0

Figure 9b shows that the myelin transmission decreases opposite the axon's transmission. When the wavelengths differ from the resonant wavelengths of the FPR, the guiding light will be reflected by the FPR and confined in the axon as the guided core mode, called ARROW^25^, while the light is launched to the axon. Output spectrum from axon output and myelin were calculated with *g-ratio* = 0.7 to study the nerve fiber as an ARROW structure. As mentioned earlier, anti-resonance modes are created when ${n}_{eff}<{n}_{Axon}$ ^18^; therefore, we have assumed ${n}_{eff}=1.0297$ ^1^.

**Figure 10:**
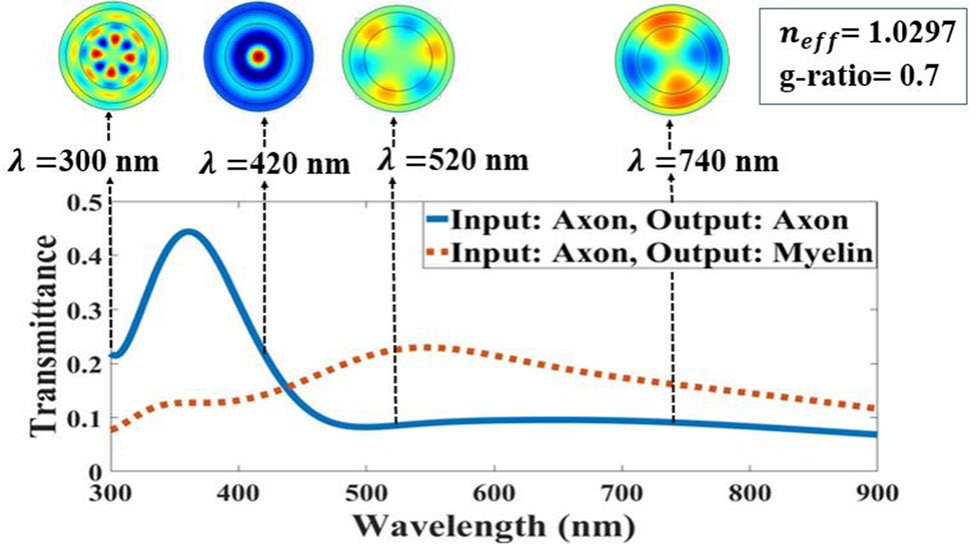
Light transmission spectra in two states of axon and myelin outputs for *g-ratio* = 0.7 with axon guided modes in 300 nm, 420 nm, 520 nm, and 740 nm wavelengths in light coupling to axon.

The transmission spectra and the conduction modes in Fig. 10 show that the maximum transmission in the axon (blue solid-line curve) occurs at low wavelengths. At higher wavelengths, the axon’s transmission decreases, and myelin (orange dotted-line curve) increases. As a result, according to the ARROW structure definition, in anti-resonance wavelength, the myelin sheath acts as an FPR and configures the light in the core or axon. According to the conduction modes in Fig. 10, they were formed inside the axons at low wavelengths and in the myelin sheath at higher wavelengths.

As shown in Fig. 10, the light transmission spectrum from the axon in the range of 300 nm—437 nm is the anti-resonance wavelength in which then axon modes are limited and satisfy the ARROW conditions in this wavelength range.

**Figure 11:**
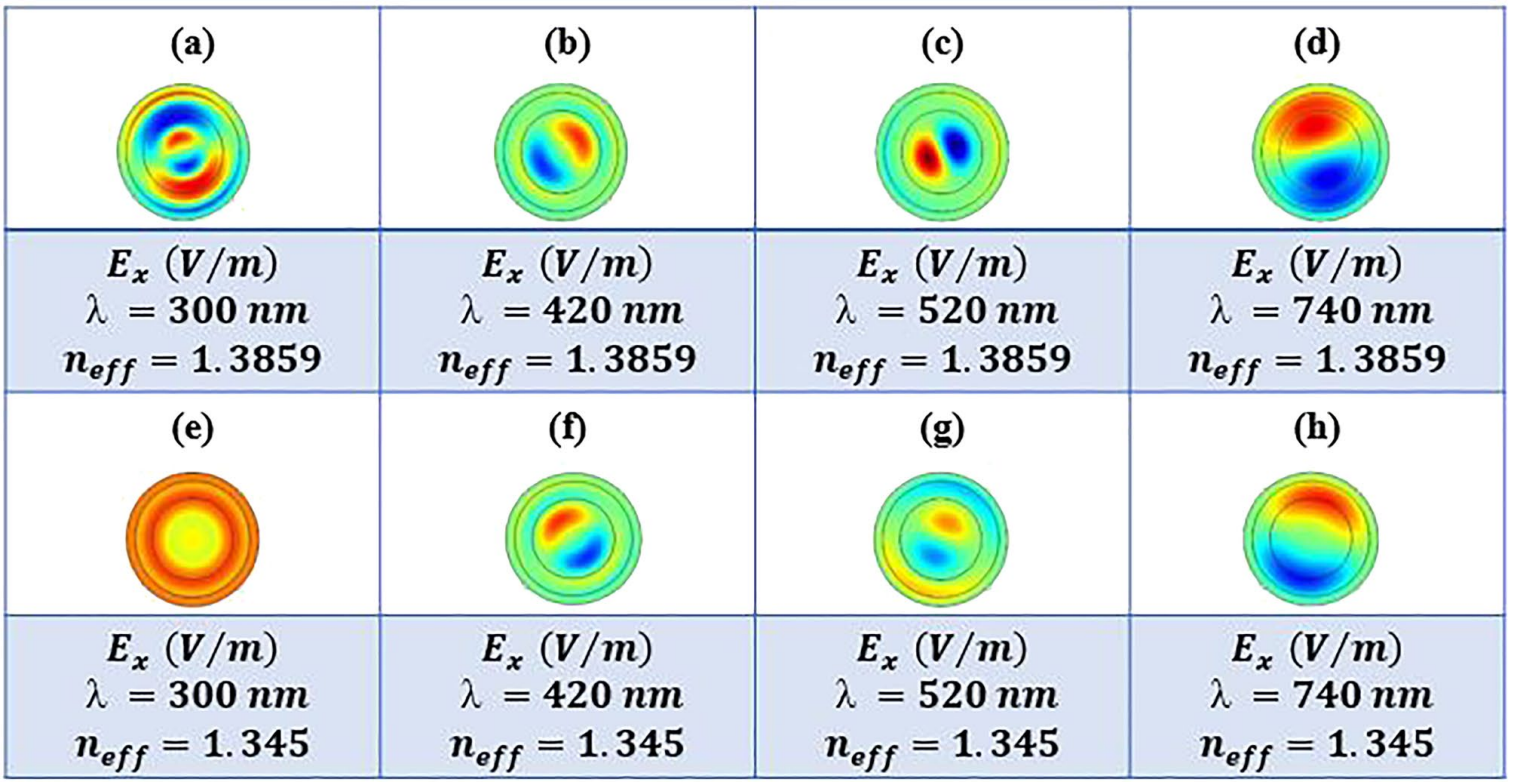
Electric field, ${E}_{x}$, distributions in nerve fiber at four different wavelengths and two different effective RI values, ${n}_{eff}$, while light is launched to the axon.

Figure 11 indicates various modes in four different wavelengths and two ${n}_{eff}$ while the light is launched to the axon. Figure 10 illustrates the nerve fiber's light transmission spectra when the conduction modes' characteristics are more inclined to be guided in the axon in the anti-resonance wavelength range.

### Bending effects and myelin sheath variation on light transmission

Myelinated axons are not necessarily straight and bend along their length^7^. In addition, the thickness of the myelin sheath is not uniform all along the length of the axon^7^; because of that, the myelin dynamically regulates its internal state in response to external physicochemical perturbations. For instance, a change in osmotic pressure induces volumetric changes in myelin, which occurs predominantly in the extracellular space^21^.

**Figure 12:**
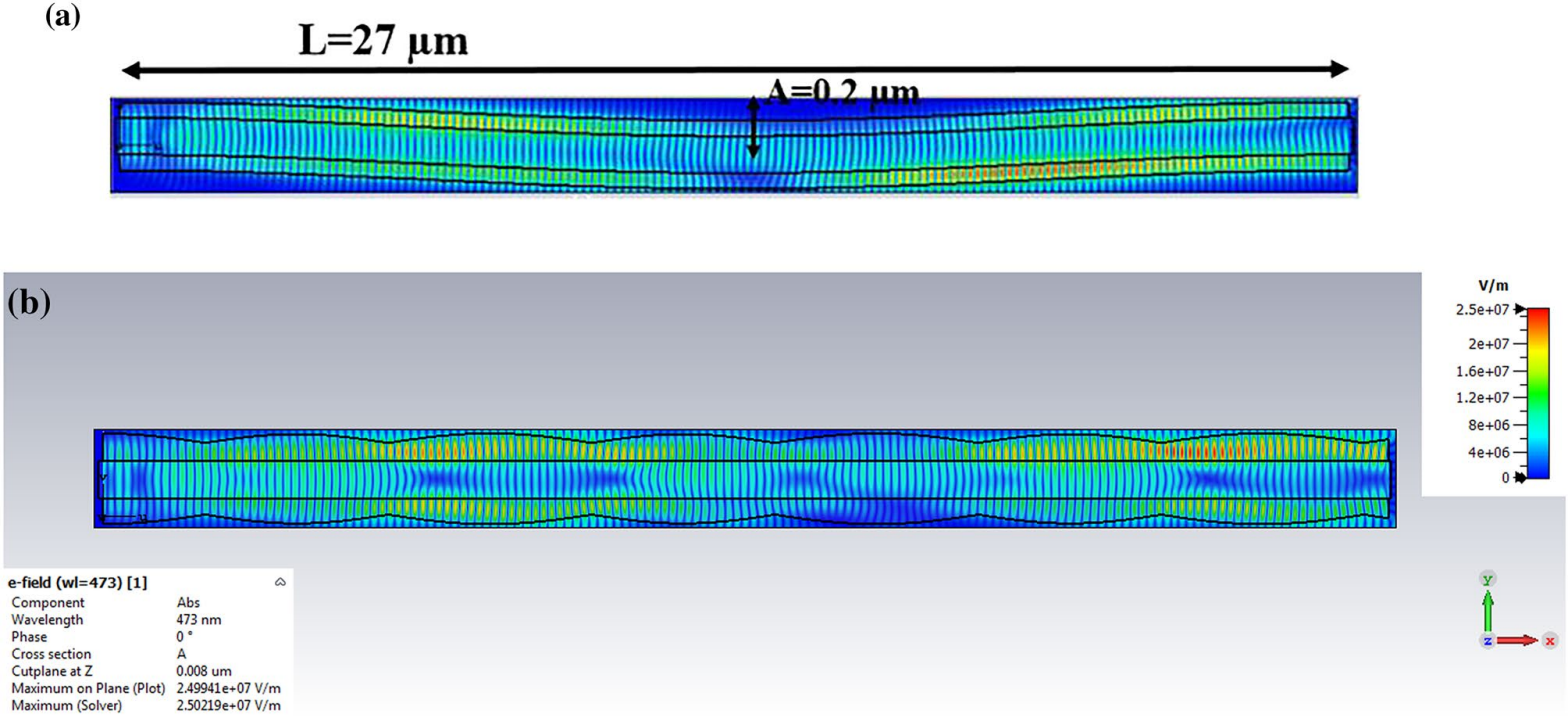
Distribution of the electric field of the myelinated axon while light is launched to the myelin sheath with a (**a**) nerve fiber with bending, and (**b**) nerve fiber with myelin sheath variation.

For this purpose, bending was defined by a curvature that $\Delta \kappa$ is a symbol of curvature. According to Ref.^7^, for $\Delta \kappa <0.05 \left({{\upmu m}}^{-1}\right),$ the transmission was greater than 90%. Besides, $\Delta \kappa$ was determined as $\Delta \kappa =4A{\left(\frac{2\pi }{L}\right)}^{2}$ in which *A* is the sinusoidal waveguide amplitude^7,27^, and *L* is the total length of a nerve fiber. Therefore, for simulation in CST software, $\Delta \kappa =0.042 \left({{\upmu m}}^{-1}\right)$ was considered, which fits in the target range ($\Delta \kappa <0.05 \left({{\upmu m}}^{-1}\right)$). Thus, according to the $\Delta \kappa$ formula and $L=27 {\upmu m}$^14^, the length of the axon, $A=0.2{\upmu m}$ was obtained. However, a cosine function like $A\mathrm{cos}\left(\frac{2\pi x}{L}\right)$, $0\le x\le L$ is needed for modeling the nerve fiber in CST software, as shown in Fig. 12a.

In addition, to show the variation in the myelin sheath, as shown in Fig. 12b, the cosine function like $A{\text{cos}}\frac{2\pi x}{{L/V}}$ is used to simulate this variation, whose amplitude and phase are assumed to be random and hypothetical^7^ where $A = 0.2{\upmu m}$ and $V = 0.3$.

Figure 12a,b show the electric field distribution of the myelinated axon in the presence of a bend and variation in the myelin sheath while the light was launched into the myelin, respectively.

**Figure 13:**
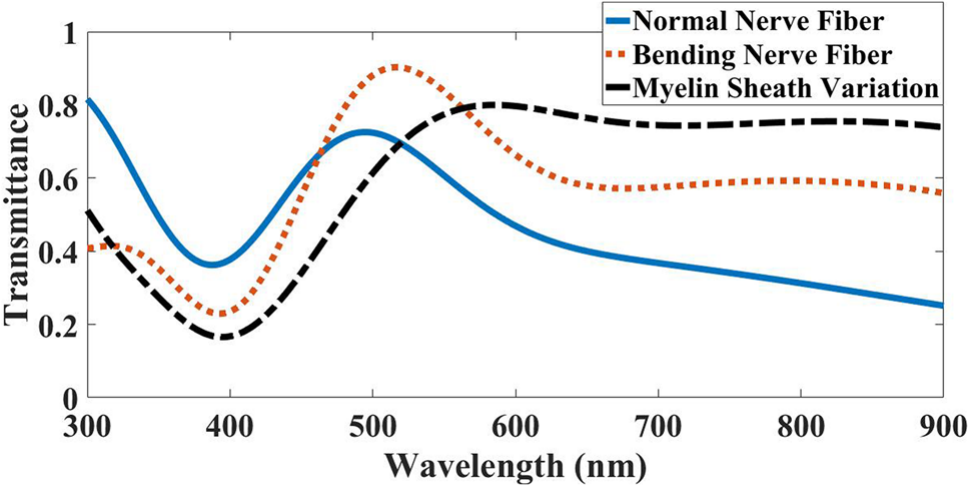
Light transmission spectra in the case of light launched to the myelin sheath in three modes of normal myelinated axons, bending nerve fiber and myelin sheath variation.

As shown in Fig. 13, the nerve fiber with bend and variation in the myelin sheath compared to the normal nerve had a blue shift in wavelength, although both follow the pattern of a normal nerve fiber (blue line). Notably, in 463 nm and 519 nm where the bending and variation transmission lines intersect the normal nerve fiber transmission line, respectively. In both cases, after these wavelengths, the transmission increased, and before them decreased. Therefore, it is concluded that after two wavelengths of 463 nm for bending and 519 nm for myelin sheath variation, the nerve fiber tends to maintain the ARROW structure and the TIR phenomenon by directing most of the modes into the myelin sheath.

These changes can be due to not following the critical angle. Besides, when the nerve fiber undergoes bending and myelin sheath variation, the angle of incident light hitting the wall of the myelin sheath will be less than the critical angle. Because of this, the light rays and guided modes will penetrate the axon^26^ and lose their light energy due to existing bend and variation in a myelin sheath^7^.

Table 4 presented the orders of magnitude transmitting for typical wavelengths in a bending nerve fiber and myelin sheath variation compared to a normal nerve.

**Table 4:** Orders of magnitude the transmission for typical wavelengths in a bending nerve fiber and myelin sheath variation compared to a normal nerve fiber when light launched to Myelin.

Imperfection	Wavelength (nm)			
	300	473	540	900
Bending nerve fiber	0.5	1.06	1.35	2.22
Myelin sheath variation	0.62	0.67	1.18	2.9

**Figure 14:**
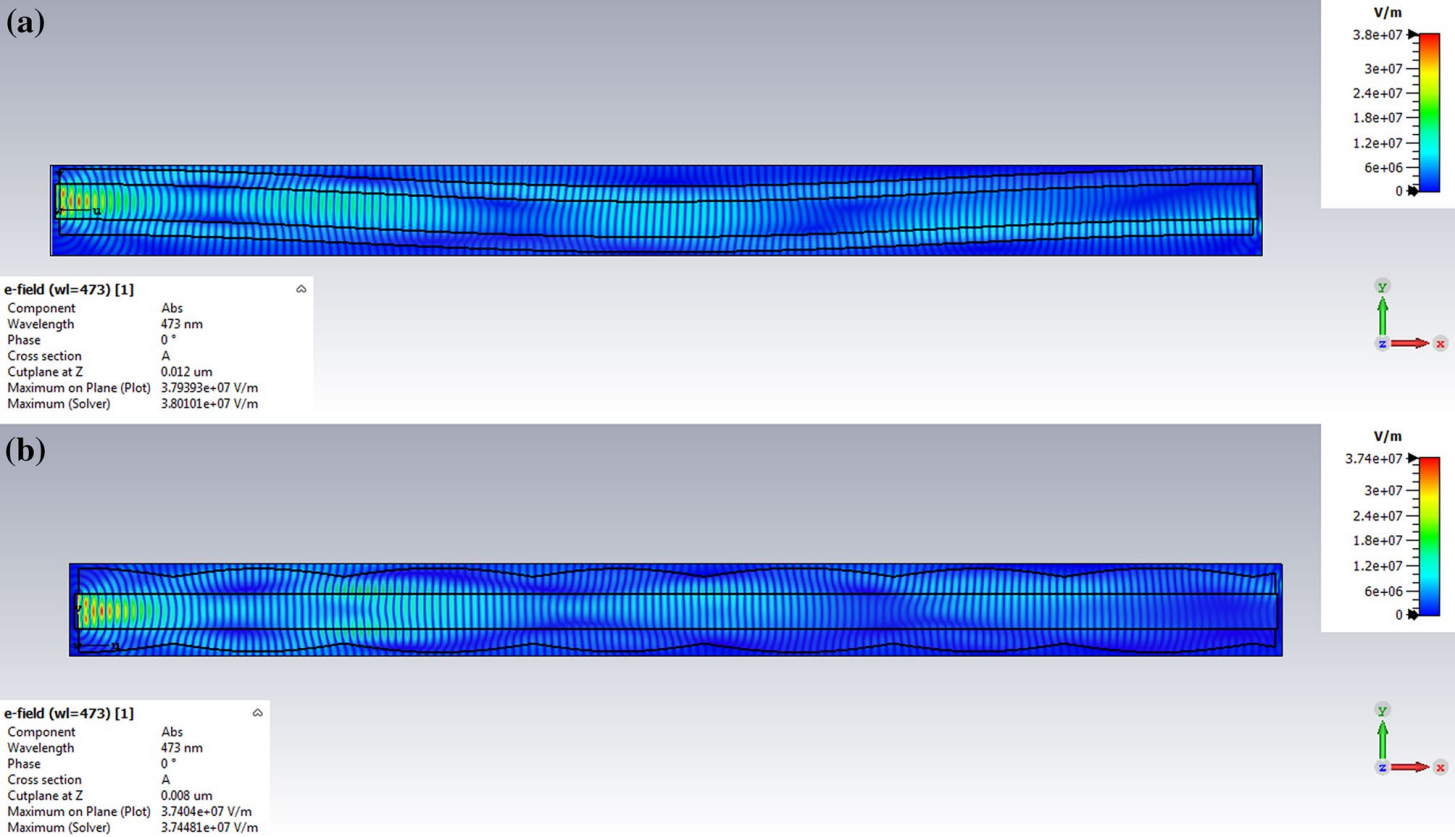
Distribution of the electric field of the myelinated axon while light is launched to the axon with (**a**) nerve fiber with bending, and (**b**) nerve fiber with myelin sheath variation.

Figure 14a,b show the distribution of the electric field with launching light to the axon in the presence of bending and myelin sheath variation, respectively. In this situation, the bends can affect the transmission by leading them outside the nerve fiber because of the critical angle^26^.

**Figure 15:**
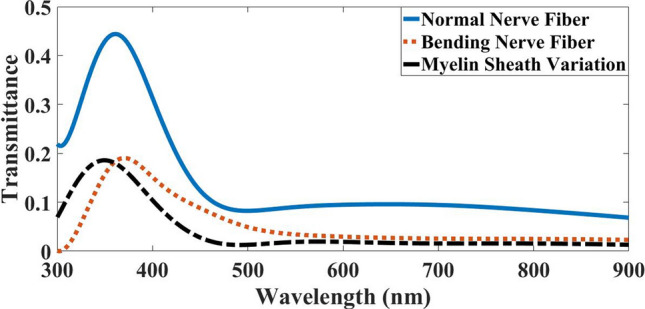
Transmission spectra in three modes of normal myelinated axon, with a bending and myelin sheath variation, while light is launched to the axon.

Figure 15 represents light transmission spectra in axons in normal nerve, with bending and myelin sheath variation. Evidently, the light transmission spectra of imperfections follow the pattern of light transmission spectra in the normal nerve fiber. Nevertheless, they had significant declines in all wavelengths, as shown in Table 5. Thus, similar to what was shown in section B and Fig. 10, the nerve fiber with bending in the range of 300 nm to 550 nm and the nerve fiber with myelin sheath variation in the range of 300 nm to 500 nm acted as an FPR. Besides, these ranges of wavelengths were also known as the anti-resonance wavelength in the ARROW structure.

**Table 5:** Orders of magnitude the transmission for typical wavelengths in a bending nerve fiber and myelin sheath variation compared to a normal nerve fiber when light launched to Axon.

Imperfection	Wavelength (nm)			
	300	473	540	900
Bending nerve fiber	0.02	0.74	0.4	0.3
Myelin sheath variation	0.3	0.16	0.2	0.18

### Effect of NR on light transmission

The NR is an integral part of myelinated axons that significantly impact action potential transfer^28^. In detail, adding the NR with the length of 0.7 µm^1^ between segments of nerve fibers in three different shapes of the normal axon, nerve fiber with bending, and myelin sheath variation, cause new modes^14^, which have impacts on light transmission in both axon and myelin.

The NR in the myelinated axon causes a crack in the myelin sheath. As a result, the modes in the NR followed Snell's law and are refracted and reflected at this point due to the difference in the RI.

**Figure 16:**
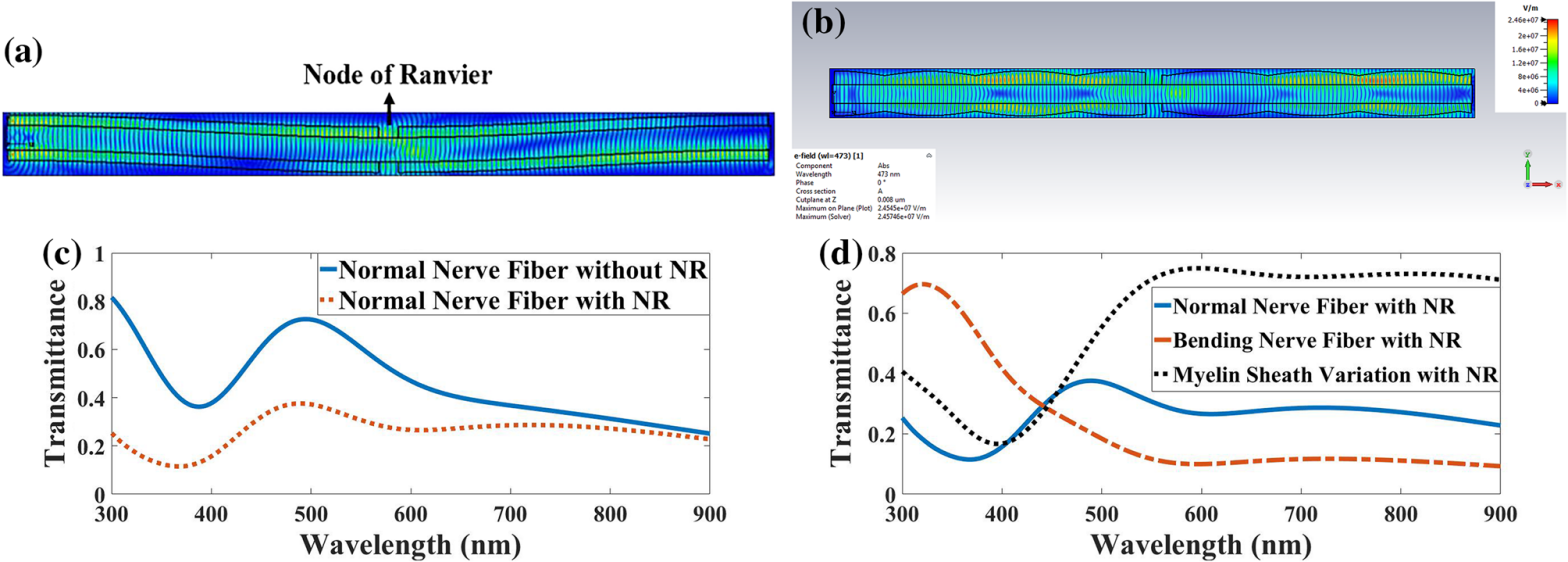
Distribution of the electric field in myelinated axon in the case of light launching to the myelin sheath in the presence of a Node of Ranvier with a (**a**) bending nerve fiber, (**b**) myelin sheath variation, (**c**) transmission spectra while light is launched to the myelin sheath in two states of with and without NR, and (**d**) transmission spectra in three modes of normal myelinated axon, nerve fiber with bending and myelin sheath variation in the presence of NR.

Figure 16a,b show electric field distribution in a bending and myelin sheath variation, respectively.

Figure 16c’s output optical spectra show the NR's effect on nerve fiber transmission. The obtained result indicated negligible changes ranging from 600 to 900 nm. Orders of magnitude of the transmission at 300 nm, 500 nm, and 700 nm in a nerve fiber with NR compared to a normal nerve fiber when the light launched into the axon were obtained at 0.31, 0.51, and 0.77, respectively. This means nerve fiber with NR in upper wavelengths preserves the TIR phenomenon.

Figure 16d compares the transmission spectra of the normal nerve fiber with bending nerve fiber and myelin sheath variation in the presence of the NR. According to Fig. 16d, the light transmission spectra for myelin sheath variation between 300 to 456 nm do not increase significantly, and only a slight blue shift occurs. However, after 456 nm, a remarkable rise was found in the transmission spectra, which means that all guided modes pass through the myelin sheath, called the TIR phenomena. Although in nerve fiber with bends and NR seems, the transmission spectra from 300 to 440 nm pass through the myelin sheath, and after 440 nm, the transmission spectra fall sharply.

This means nerve fibers with bend and NR act as an ARROW structure only between 300 to 440 nm. These changes in the amount of transmission shown in Table 6 would be due to the constructive interference of the leaky modes and conduction modes in the NR^22,23^ and not following the critical angle^26^.

**Table 6:** Orders of magnitude the transmission for typical wavelengths in a bending nerve fiber and myelin sheath variation with NR compared to a normal nerve fiber when light launched to Myelin.

Imperfection	Wavelength (nm)			
	300	473	540	900
Bending nerve fiber with NR	2.6	0.6	0.4	0.4
Myelin sheath variation with NR	1.6	1.15	2.1	3.1

**Figure 17:**
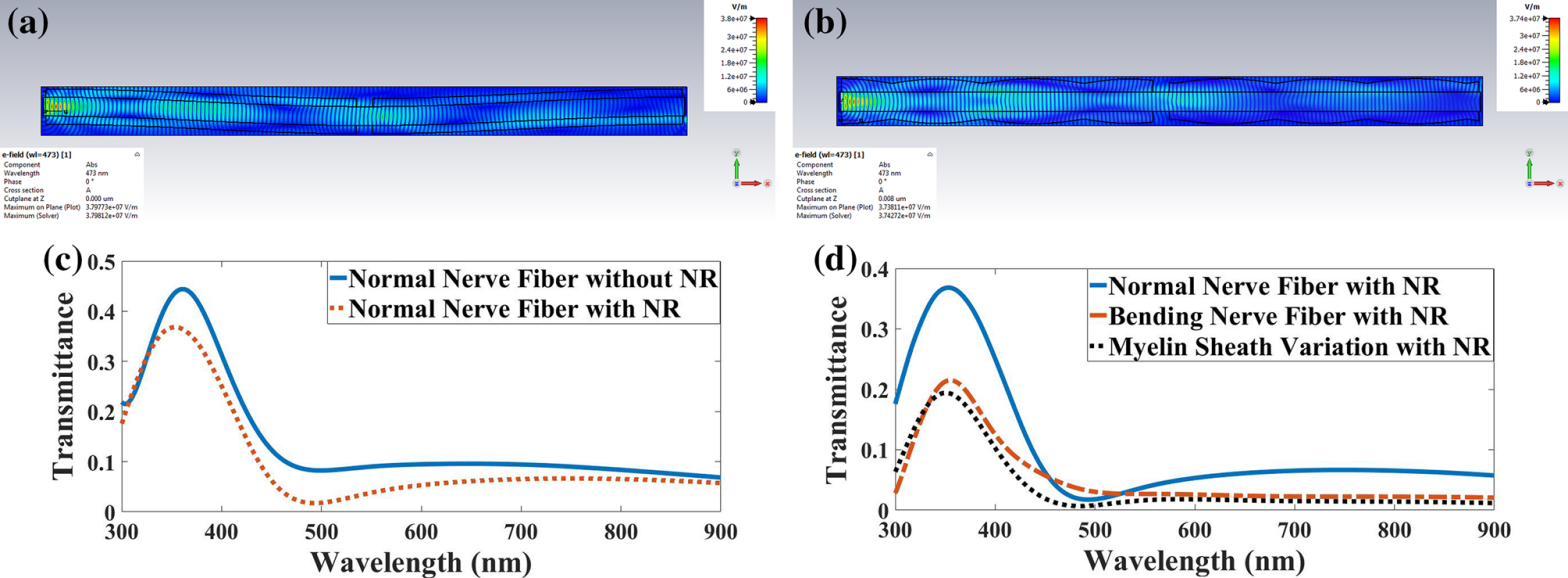
Distribution of the electric field in the myelinated axon with a NR when light is launched to the axon with a (**a**) bending, (**b**) myelin sheath variation. Transmission spectra in normal myelinated axon with (**c**) NR, and (**d**) a bending and myelin sheath variation.

Demonstratively, the NR is an integral part of the myelinated axon affecting light transmission^14^. Figure 17a,b show the distribution of the electric field in both bending and myelin sheath variation in the nerve fiber with NR while the light was launched to the axon.

Figure 17c shows that the transmission decreased in normal fiber with the NR because of the light refraction and reflection in the NR. Table 7 presented the orders of magnitude of the transmission at 360 nm, 500 nm, and 700 nm in a nerve fiber with NR compared to a normal nerve fiber when the light was launched into the axon obtained at 0.8, 0.2, and 0.6, respectively. However, Fig. 17d shows that due to the launching of light into the axon, considerable declines were found in both bending nerve fiber and myelin sheath variation with NR. Thus, in this case, the nerve fiber acted as an ARROW only in the wavelength ranging from 300 to 500 nm, known as anti-resonance wavelengths.

**Table 7:** Orders of magnitude the transmission for typical wavelengths in a bending nerve fiber and myelin sheath variation compared to a normal nerve fiber when light launched to Axon.

Imperfection	Wavelength (nm)			
	300	473	540	900
Bending nerve fiber with NR	0.15	1.6	0.8	0.36
Myelin sheath variation with NR	0.36	0.3	0.4	0.2

The arguments revealed above suggest that despite many questions that are yet unanswered, our approach, together with the optical guiding system hypotheses, not only conforms to the possible involvement of photons in neuronal signaling but also contributes to disclosing their possible role in integrating other known mechanisms for information transmission in the myelinated axon. In addition to its conventional role as an insulator, the myelin sheath might also have the ability to act as an optical waveguide, which may help us to understand better the causes of the diseases associated with it as well as the subsequent treatment requirement and design^7^.

## Conclusion

In this study, it has been shown that nerve fiber can act as a waveguide, and the condition of light launching to the axon or myelin sheath can determine the physical phenomenon of light transmission inside the nerve fiber.

This idea originated from the fact that the refractive index (RI) profile of a nerve fiber is not the same as a step-index optical fiber but different from it and similar to the RI profile of depressed core fibers (DCFs) fibers. This difference creates a new feature called an anti-resonant reflecting optical waveguide (ARROW).

Assumedly, to prove the hypothesis, the myelinated axon acts similarly to DCF, and the light was launched in two cases of the myelin sheath and axon to expand this idea and examine the behavior of the nerve fiber in different conditions. In the first case, when the light is launched into the myelin sheath, it is observed that the conduction modes are more inclined to pass through the myelin sheath. The ideal state is when *g-ratio* = 0.7, where incident light passes through the myelin, and the light passing through the axon is zero. The shape of the conduction modes obtained at different wavelengths and RIs confirms this. According to Ref^18^. and the obtained results, it can be concluded that resonant wavelengths are activated by launching the light to the myelin sheath, and the total internal reflection (TIR) phenomenon occurs. In the second step, light is launched into the axon, and obtained results showed that more conduction modes pass through the axon in the wavelength range of 300 nm to 433 nm. According to the conduction modes, light transmission spectra, and the results of Ref.^18^ and Ref.^25^, it can be concluded that these wavelengths are anti-resonance in the ARROW structure. Because of this, the myelin sheath acts as a Fabry–Perot resonator (FPR) in this wavelength range to confined conduction modes to the axon.

Evidently, nerve fiber is not straight along its length and has bend^7^, the myelin sheath may be varied due to changes in osmotic pressure^7,21^, and the NR is an inseparable part of the myelinated axon. Therefore, the presence of these imperfections can affect the results of the hypothesis. When light is launched into myelin, the intensity of light transmission increases in the wavelength ranging from 463 to 900 nm when the nerve fiber is bending and 519 nm to 900 nm when the myelin sheath variation. Because of these wavelengths, the more guided modes pass through the myelin sheath, and as a result, the TIR phenomena happen perfectly. For example, at 540 nm, the light intensity in bending and myelin sheath variation is 1.35 and 1.18 times more than the light intensity of normal nerve fiber.

While in the launching light to the axon, the anti-resonance wavelengths appear in lower wavelengths. This means that, in the presence of bending and myelin sheath variation, the wavelength ranging from 300 to 500 nm and 300 nm to 550 nm acts as anti-resonance wavelengths due to most guided modes passing through the axon. In this situation, the myelin sheath act as an FPR in the ARROW structure. For example, at 473 nm, the light intensity in bending and myelin sheath variation is 0.74 and 0.16 times less than the light intensity of normal nerve fiber.

The next step was to consider the bending and variation of the myelin sheath with the presence of NR. In the first case, when the light is launched into the myelin sheath, the wavelength ranging from 300 to 440 nm shows the highest transmission in the myelin sheath in the presence of bend and NR, while in the wavelength range of 456 nm to 900 nm shows the most significant amount of light transmission in the presence of myelin sheath variation and NR. In other words, these two ranges of wavelengths are suitable for the occurrence of the TIR phenomenon. For example, the light intensity in nerve fiber with bending and NR at 300 nm NR is 2.6 times more than the light intensity of normal nerve fiber with NR, and in variation, myelin sheath with NR at 540 nm is 2.1 times more than the light intensity of normal nerve fiber with NR. In conclusion, when the light is launched into the axon, a significant reduction can be seen in the wavelength range of 300 nm to 540 nm in both the bending and the myelin sheath variation with NR. Despite these reductions, most light intensity passes through the axon in this wavelength range. This range should be known as an anti-resonance wavelength in the ARROW structure. For example, at 540 nm, the light intensity in bending and myelin sheath variation with NR is 0.8 and 0.4 times less than the light intensity of normal nerve fiber with NR.

The reason for these changes in different modes is that the light beam does not follow the critical angle for effective transmission in the presence of bending and variation^26^. Besides, the NRs in the nerve fiber are like cracks, and variations of the environment and its refractive index (RI) cause changes in the speed and direction of light rays in the area^29,30^. Hence, the existing modes can have constructive or destructive interference, in which the intensity of the light transmission^14^ is increased or reduced.

This approach can be an optical model for a neural cell that provides the basis for new studies in the field of cellular messaging and deep brain stimulation based on the concepts of nanophotonic communication. The study of optical properties of neurons based on ARROW structure can be used to study neural cell structure in the early stages of diagnosing neurological diseases such as multiple sclerosis (MS). Also, understanding the optical behavior of neural cells at the micrometer scales can be used in optical stimulation techniques based on optogenetic techniques, brain-on-a-chip, or optical modulation of nervous systems. The waveguiding properties of nerve cells can be used to develop optical equipment based on biological structures such as optical switches and bio-lasers.

## Data Availability

Data underlying the results presented in this paper are not publicly available at this time but may be obtained from the authors upon reasonable request.
